# Prevalence and correlates of alcohol use and risky drinking among undergraduate students in Johannesburg, South Africa: a cross-sectional study

**DOI:** 10.1186/s12888-023-05043-w

**Published:** 2023-08-01

**Authors:** Cassandra Chen, Mafuno G Mpinganjira, Asha Motilal, Sandile Matukane, Relebohile Letsoalo, Tyler McKee, Zakithi Ntombela, Limuwani Mbulaheni, Taveer Hargovan, Joel M Francis

**Affiliations:** 1 School of Clinical Medicine, Faculty of Health Sciences, UUME, University of the Witwatersrand, Johannesburg, South Africa; 2 Department of Family Medicine and Primary Care, School of Clinical Medicine, Faculty of Health Sciences, University of the Witwatersrand, Johannesburg, South Africa

**Keywords:** Alcohol use, Alcohol use disorder, Alcohol abuse, Risky drinking, Substance abuse, Youth.

## Abstract

**Background:**

Alcohol use and risky drinking are significant public health problem globally. Young people, including university students, are among the most affected populations. We conducted the study to determine the prevalence and correlates of alcohol use and risky drinking among undergraduate students in the Faculty of Health Sciences at the University of the Witwatersrand, South Africa.

**Methods:**

We conducted a cross-sectional study using an anonymous, self-administered online survey in REDCap. The survey questionnaire consisted of socio demographic, and alcohol use questions using the risky drinking identification screening tool (AUDIT-C). We performed descriptive statistics, bivariate and multivariable logistic regression to determine factors associated with alcohol use and risky drinking. The p-value of < 0.05 was considered statistically significant.

**Results:**

The response rate was 15.7%. Most participants were female (69.6%) and majority of the participants were White (38.1%). The prevalence of lifetime use of alcohol was 79.1%, and among the lifetime users; 70.2% reported alcohol use in the last 12-months, 37.1% reported alcohol use in the last 30 days. The prevalence of risky drinking was 54.8% among lifetime drinkers. Factors significantly associated with current alcohol use were siblings alcohol use (aOR = 1.79, 95% CI: 1.02–3.15) and parents alcohol use (aOR = 2.58, 95% CI: 1.39–4.80), white race (aOR = 5.70, 95% CI: 3.12–10.41), and always or daily exposure to alcohol marketing in the media (aOR = 3.31, 95% CI: 1.07–10.24). Factors associated with risky drinking were: Indian/Asian race (aOR = 2.82, 95% CI: 1.09–7.31), White race (aOR = 2.15, 95% CI: 1.14–4.04), and exposure to alcohol marketing in the media as follows, most of the time (aOR = 3.42, 95% CI: 1.29–9.04) and Always/daily exposure (aOR = 3.31, 95% CI: 1.07–10.24).

**Conclusion:**

The reported alcohol use and risky drinking were common amongst undergraduate students at Wits university. There is an urgent need to design, pilot and adapt targeted interventions for this population group.

**Supplementary Information:**

The online version contains supplementary material available at 10.1186/s12888-023-05043-w.

## Background

Excessive alcohol use is a significant public health problem globally. According to WHO; harmful alcohol use was responsible for 3 million deaths and 132.6 million disability-adjusted life years (DALYs) – 5.3% of all deaths and 5.1% of all DALYs in 2016 [1]. In sub-Saharan Africa (SSA), alcohol use is a leading risk factor for both death and disability [2]. Previous studies on alcohol use in Africa showed that specific groups of young people, sex workers and university/college students, reported high rates of alcohol consumption [3, 4].The existing alcohol literature reported sex, belonging to age band 15–29 years, disposable income, peer pressure, exposure to advertisement, multiple substance use, family and siblings’ alcohol use, living arrangement, for example, staying on campus, religion and religiosity are associated with alcohol use and alcohol use disorders (AUDs) [1, 5]. Several factors have been associated with alcohol consumption in young people [1]. Males, those within the age group of 15–29 years, disposable income, and being university and college students are key drivers to alcohol use and alcohol use disorder (AUD) among young people [1, 4, 6]. South Africa is among the countries with the highest rates of harmful alcohol use patterns in the world [5].

There is increased alcohol marketing and promotion in Africa, which has led to a predicted increase in alcohol sales in the continent [7]. In a study conducted on adolescents in the Tshwane metropolitan, South Africa, it was found that the prevalence of alcohol consumption in the past 6 months was greatest in those who reported exposure through media platforms such as TV programs and films and receiving emails with advertisements and promotions, as well as promotion of alcohol brands by famous people and free offers when they bought alcohol [8, 9]. Peer pressure, exposure to alcohol advertisements, other substance use, sibling or parental alcohol use [1, 10], and staying on campus/living unsupervised [11] were associated with alcohol use and AUD. On the other hand, higher religiosity and being a Muslim have been identified as protective factors to harmful alcohol use and AUD [12–14].

Despite alcohol use and AUD being common in SSA, evidence shows that there are few policy and individual level interventions [6, 15–17]. There are however a few reported efforts to address harmful alcohol use such as the work of van der Westhuizen et al., 2019 in emergency health centres in Western Cape, South Africa that evaluated the feasibility of adaptation of Screening, Brief Intervention, and Referral to Treatment (SBIRT) for risky alcohol use in routine services [18] but studies are lacking on SBIRT among university students in South Africa and other settings in sub-Sahara Africa, except for an ongoing effort in Kenya by Musyoka et al., 2020 on mobile health intervention on substance use among first university undergraduate students [19].

Previous studies in South Africa have reported on the prevalence and factors associated with alcohol use among university students in South Africa and most of these studies were conducted in less than half of the total number of provinces in the country namely; Limpopo, Western Cape, Eastern Cape, and Free State [6, 10, 20, 21]. There is limited data on alcohol use and risky drinking among university students in Gauteng province; the most dynamic, populous, and economic hub of South Africa – a province which also hosts the executive government of the country. Understanding the prevalence and factors associated with alcohol use among university students in Gauteng province is critical and will inform potential interventions in this high-risk population for alcohol use and risky drinking.

This study determined the prevalence and factors associated with alcohol use and risky drinking among university students in South Africa – a sample that was conveniently selected.

## Methods

### Study design and population

We conducted a cross-sectional survey among undergraduate students registered at the Faculty of Health Sciences (FHS) at the University of the Witwatersrand (WITS), Johannesburg, South Africa, from November 2019 to February 2020.

### Sample size and sampling

The undergraduate cohort consisted of 4016 students in the FHS. The minimum sample size was calculated using OpenEpi version 3.01 [22] with the following assumptions: the proportion of students who used alcohol in the past 12 months was 50% and the proportion of risky drinking among those who use alcohol was 50% [20]. An anticipated percentage frequency of 50% was thus used with a 5% margin of error and a design effect of 2. The estimated minimum sample size was 702, however, because we expected that the response rate would be low and to account for an assumed nonresponse rate of 50%, the final target minimum sample size was adjusted to 1053 students. All undergraduate students in the FHS were eligible and invited to participate in the study.

### Data collection procedures

Data was collected using an electronic standardised 35-item English questionnaire (AUDIT-C) embedded into REDCap. A link with the questionnaire, information sheet, the consent form and the questionnaire were shared with the University Registrar’s office for sharing with the students registered at FHS on behalf of the study team. All participants voluntarily responded to the questionnaire after providing informed consent. Due to the anticipated nonresponse rate, a reminder email to participate in the study was sent out two weeks after the commencement of data collection. All responses were stored in the password protected REDCap cloud server accessed only by the study team and the supervisor.

### Ethical considerations

The study received ethics approval from Wits Health Research Ethics Committee in October 2019, reference number: M1909102. Permission to conduct the study was obtained from the university authorities (registrar’s office and respective heads of schools). All participants provided a voluntary informed consent prior to responding to the online survey anonymously. The responses were stored in a secured cloud server.

### Study variables

#### Outcomes

The primary outcomes of interest were reported alcohol use – defined as (lifetime use, past 12-month use, and past 30-day use), and risky drinking – assessed by a standardised and validated alcohol screening questionnaire among young people in Africa – the AUDIT-C [23, 24]. The AUDIT-C scores ranges from 0 to 12, the risky drinking was defined as participants scoring ≥ 3 for women and ≥ 4 for men.

The secondary outcome of interest was the preferred approaches to alcohol interventions programmes.

#### Exposures

Exposures were sociodemographic factors which included programme of study, year of study, gender, age, race, religion, marital status, income level, and living arrangements of the study participants. Race in South African contexts, the terms “white”, “black”, and “Coloured”, originate from the apartheid era. They refer to demographic markers and do not signify inherent characteristics. They refer to people of European, African and mixed (African, European and/or Asian) ancestry, respectively. These markers were chosen for their historical significance. Their continued use in South Africa is important for monitoring improvements in health and socio-economic disparities, identifying vulnerable sections of the population, and planning effective prevention and intervention programmes.

### Data management and analysis

We used STATA version 14 to clean and analyse the survey data. Descriptive statistics, mainly proportions, were computed for the categorical variables. A bivariate analysis was used to determine the association between reported alcohol use/risky drinking and exposures using the Chi^2^ test. The results from the bivariate Chi^2^ analyses were used to indicate which variables were eligible for inclusion in the multivariable logistic regression models. Multivariable logistic regressions were run to determine factors associated with lifetime, past 12-month, past 30-day alcohol use and risky drinking. Age and sex were considered *a priori* as potential confounders to alcohol use and risky drinking [3] and included in the multivariable models regardless of their p values in the bivariate analysis. Other variables were included in the multivariable models if they had a p-value < 0.20 from the bivariate analysis [25], (Supplementary Table 1). Findings from multivariable models were reported as adjusted odds ratios (aOR). We further performed a sensitivity analysis to determine the difference in characteristics between individuals who completed the survey versus those who did not A p-value of < 0.05 was considered statistically significant.

## Results

Out of the 631 who opened the survey link, 92 did not complete the questionnaire thus only 539 (85.4%) were included in the analysis. Socio demographic characteristics of participants who completed the survey only differed from those who partially completed the survey by ‘living situation’ (Supplementary Table 2).

### Study Population characteristics

Of the 538 students whose data were analysed, majority were female (n = 374, 69.6%). Most participants (n = 370, 68.8%) were medical students enrolled into the MBBCh degree and about half of the participants (n = 237, 44.1%) belonged to 20–23 years age band. Christianity was the most common reported religion (n = 311, 57.8%). Majority of the participants were single (n = 390, 72.5%). The reported median monthly income was R2000, equivalent to 133USD. Almost half of the study participants (n = 260, 48.3%) reported living with an adult (Table 1).

**Table 1:** Sociodemographic profile of WITS Faculty of Health Sciences undergraduate students, Johannesburg, South Africa, 2019–2020

Characteristic	Categories	n	%
Gender	Female	374	69.6
	Male	163	30.4
	Total	537	100.0
Programme of study	MBBCh^1^	370	68.8
	Other	168	31.2
	Total	538	100.0
Current year of study	1	104	19.3
	2	79	14.7
	3	116	21.6
	4	117	21.7
	5	59	11.0
	6	63	11.7
	Total	538	100.0
Racial identity^2^	Black African	183	35.1
	Coloured	25	4.8
	Indian/Asian	115	22.0
	White	199	38.1
	Total^3^	522	100.0
Age (in years)	Less than 20	199	37.0
	20–23	237	44.1
	Greater than 24	102	19.0
	Total	538	100.0
Religion	Christian	311	57.8
	Muslim	71	13.2
	Atheist/Agnostic	90	16.7
	Other	66	12.3
	Total	538	100.0
Marital status	Single	390	72.5
	In a relationship	148	27.5
	Total	538	100.0
Income (median = R2,000)	Less than or equal to median	326	60.6
	Greater than median	212	39.4
	Total	538	100.0
Living situation	Living with an adult	260	48.3
	Living alone	266	49.4
	Other	12	2.2
	Total	538	100.0

^1^ MBCHB (Bachelor of Medicine and Bachelor of Surgery), ^2^ Race categories recognized in South Africa, ^3^Varying total because of missing values

### Prevalence of alcohol use and risky drinking

The prevalence of lifetime use of alcohol was (n = 425, 79.1%), (n = 377, 70.2%) for past 12-month alcohol use, and (n = 199, 37.1%) for past 30-day alcohol use. The prevalence of risky drinking as defined by AUDIT-C was (n = 206, 54.8%). The prevalence of risky drinking was 54.3% and 56.0% in Females and Males respectively. (Table 2).

**Table 2:** Patterns of alcohol use among the WITS Faculty of Health Sciences undergraduate students, Johannesburg, South Africa, 2019–2020

Characteristic	Categories	Female		Male		Total^d^	
		n	%^a^	n	%^a^	n	%^a^
Lifetime alcohol use	No	69	18.4	43	26.4	112	20.9
	Yes	305	81.6	120	73.6	425	79.1
	Total	374	100.0	163	100.0	537	100.0
Past 12-month alcohol use	No	105	28.1	55	33.7	160	29.8
	Yes	269	71.9	108	66.3	377	70.2
	Total	374	100.0	163	100.0	537	100.0
Past 30-day alcohol use	No	232	62.0	106	65.0	338	62.9
	Yes	142	38.0	57	35.0	199	37.1
	Total	374	100.0	163	100.0	537	100.0
Frequency of having drink containing alcohol	Never	4	1.5	3	2.8	7	1.9
	Monthly or less	144	53.7	56	51.4	200	53.1
	2–4 times a month	96	35.8	34	31.2	130	34.5
	2–3 times a week	22	8.2	13	11.9	35	9.3
	4 or more times a week	2	0.7	3	2.8	5	1.3
	Total	268	100.0	109	100.0	377	100.0
Total number of standard drinks on a typical day when drinking	1 or 2	131	48.9	37	33.9	168	44.6
	3 or 4	109	40.7	27	24.8	136	36.1
	5 or 6	20	7.5	21	19.3	41	10.9
	7 or 9	5	1.9	15	13.8	20	5.3
	10 or more	3	1.1	9	8.3	12	3.2
	Total	268	100.0	109	100.0	377	100.0
How often do you have six or more drinks on one occasion?	Never	129	48.0	27	24.8	156	41.3
	Less than monthly	118	43.9	51	46.8	169	44.7
	Monthly	18	6.7	27	24.8	45	11.9
	Weekly	4	1.5	3	2.8	7	1.9
	Daily or almost daily	0	0.0	1	0.9	1	0.3
	Total	269	100.0	109	100.0	378	100.0
Risky drinking according to AUDIT-C^b^	No	122	45.7	48	44.0	170	45.2
	Yes	145	54.3	61	56.0	206	54.8
	Total	267	100.0	109	100.0	376	100.0
Occasion of first alcoholic drink	Holiday	28	9.2	14	11.7	42	9.9
	Wedding ceremony	7	2.3	3	2.5	10	2.4
	School party/graduation	10	3.3	15	12.5	25	5.9
	Going out/at a party with friends	152	50.0	47	39.2	199	46.9
	Other family celebration	83	27.3	33	27.5	116	27.4
	Other	24	7.9	8	6.7	32	7.5
	Total	304	100.0	120	100.0	424	100.0
Type of alcoholic drink on first drinking occasion	Bottled beer	37	12.1	45	37.5	82	19.3
	Wine	77	25.2	15	12.5	92	21.6
	Spirit/liquor	145	47.5	54	45.0	199	46.8
	Home-brewed spirit/liquor	6	2.0	1	0.8	7	1.6
	Other	40	13.1	5	4.2	45	10.6
	Total	305	100.0	120	100.0	425	100.0

^a^ Column percentages ^b^ Participants included in analysis are those with past 12-month alcohol use, ^d^ Varying totals because of missing values

### Factors associated with alcohol use and risky drinking

From the multivariable analyses participants who identified as being of the white race were associated with increased odds of lifetime use of alcohol (adjusted Odds Ratio [aOR] = 3.41, 95% CI: 1.15–10.17), past 12-month use of alcohol (aOR=: 3.46, 95% CI: 1.65–7.23) and past 30-day use of alcohol (aOR: 5.70, 95% CI: 3.12–10.41) as compared to students who identified as Black African. On the other hand, identifying as Indian/Asian (aOR:2.82, 95% CI: 1.09–7.31) or White (aOR = 2.15, 95% CI: 1.14–4.04) was associated with risky drinking as compared to students identified as Black African. Having any parents who drank alcohol was associated with increased odds of lifetime use of alcohol (aOR = 4.56, 95% CI: 2.06–10.09), past 12-month use of alcohol (aOR = 4.32, 95% CI: 2.34–7.97), past 30-day use of alcohol (aOR = 2.58, 95% CI: 1.39–4.80). Furthermore, having siblings who drank alcohol was associated with higher odds of past 12-month use of alcohol (aOR = 2.66, 95% CI: 1.40–5.04) and past 30-day use of alcohol (aOR = 1.79, 95% CI: 1.02–3.15). Exposure to alcohol adverts (always or daily) was associated with reporting alcohol use in the last 30 days (aOR = 3.31, 95% CI, 1.07–10.24) and risky drinking (aOR = 4.00, 95% CI: 1.43–11.20). Income greater than median (133 USD) was associated with lifetime use of alcohol (aOR = 2.40, 95% CI: 1.01–5.68).

On the contrary, being Muslim, decreased the odds of lifetime use of alcohol (aOR = 0.03, 95% CI: 0.01-0.013) and past 12-month use of alcohol (aOR = 0.03, 95% CI: 0.01–0.13). (Table 3).

**Table 3:** Factors associated with alcohol-use and alcohol-use-disorder among the WITS Faculty of Health Sciences undergraduate students

		Lifetime alcohol use			Past 12-month alcohol use			Past 30-day alcohol use			Risky drinking ^b^		
Characteristic	Categories	AOR^a^	95% CI	P-value	AOR^a^	95% CI	P-value	AOR^a^	95% CI	P-value	AOR^a^	95% CI	P-value
Gender	Female	1.00			1.00			1.00			1.00		
	Male	0.60	0.28–1.28	0.186	0.84	0.45–1.56	0.584	0.85	0.50–1.46	0.561	0.75	0.43–1.28	0.287
Programme of study	MBCHB	1.00			1.00			1.00			1.00		
	Other	1.21	0.51–2.85	0.662	2.01	0.99–4.08	0.054	0.88	0.49–1.58	0.664	1.03	0.57–1.88	0.921
Current year of study	1	1.00			1.00			1.00			1.00		
	2	2.80	0.82–9.58	0.101	1.42	0.53–3.78	0.489	1.04	0.44–2.45	0.924	0.85	0.37–1.95	0.696
	3	1.69	0.44–6.49	0.442	1.38	0.45–4.18	0.571	1.17	0.45–3.09	0.745	0.43	0.16–1.18	0.103
	4	1.54	0.35–6.83	0.569	1.34	0.40–4.52	0.683	0.74	0.25–2.17	0.588	0.42	0.14–1.27	0.124
	5	0.39	0.07–2.32	0.301	1.01	0.23–4.45	0.993	0.66	0.18–2.35	0.519	0.57	0.15–2.14	0.407
	6	7.77	0.60–99.97	0.116	4.25	0.77–23.54	0.097	1.76	0.47–6.55	0.397	0.74	0.20–2.72	0.648
Racial identity	Black African	1.00			1.00			1.00			1.00		
	Coloured	2.39	0.39–14.76	0.348	3.63	0.74–17.86	0.113	1.37	0.38–4.95	0.632	0.94	0.27–3.31	0.928
	Indian/Asian	0.87	0.26–2.97	0.829	2.86	0.91–9.01	0.072	2.39	0.97–5.91	0.059	2.82*	1.09–7.31	0.033
	White	3.41*	1.15–10.17	0.023	3.46***	1.65–7.23	0.001	5.70***	3.12–10.41	< 0.001	2.15*	1.14–4.04	0.017
Age in years	Less than 20 years	1.00			1.00			1.00			1.00		
	20–23 years	0.71	0.21–2.37	0.578	0.45	0.18–1.15	0.095	1.06	0.46–2.44	0.884	2.28	0.94–5.51	0.067
	Above 24 years	3.14	0.54–18.43	0.205	0.55	0.16–1.91	0.35	1.04	0.36–3.01	0.94	1.81	0.61–5.35	0.286
Religion	Christian	1.00			1.00			1.00			1.00		
	Muslim	0.03***	0.01–0.13	< 0.001	0.03***	0.01–0.13	< 0.001	-	-		0.83	0.06–11.75	0.894
	Atheist/Agnostic	1.94	0.51–7.43	0.333	2.43	0.87–6.81	0.092	1.68	0.87–3.24	0.122	1.84	0.96–3.50	0.064
	Other	1.60	0.42–6.10	0.494	1.50	0.52–4.37	0.453	1.23	0.57–2.66	0.607	1.65	0.75–3.64	0.213
Marital status	Single	1.00			1.00			1.00			1.00		
	In a relationship	1.75	0.63–4.83	0.283	1.03	0.51–2.09	0.935	1.22	0.71–2.10	0.467	1.11	0.64–1.91	0.721
Income (median = R2,000)	Equal to or below median	1.00			1.00			1.00			1.00		
	Greater than median	2.40*	1.01–5.68	0.047	1.79	0.92–3.49	0.086	1.59	0.93–2.70	0.089	1.00	0.58–1.72	0.993
Living arrangement	Living with an adult	1.00			1.00			1.00			1.00		
	Living alone	1.29	0.60–2.80	0.515	1.47	0.80–2.73	0.218	1.37	0.80–2.32	0.248	1.54	0.90–2.62	0.116
	Other	0.84	0.07–9.63	0.89	0.56	0.09–3.43	0.53	0.73	0.14–3.72	0.707	0.34	0.05–2.21	0.258
Have any siblings who drink alcohol	No	1.00			1.00			1.00			1.00		
	Yes	1.89	0.84–4.23	0.125	2.66**	1.40–5.04	0.003	1.79*	1.02–3.15	0.042	1.20	0.68–2.13	0.533
Have any parents who drink alcohol	No	1.00			1.00			1.00			1.00		
	Yes	4.56***	2.06–10.09	< 0.001	4.32***	2.34–7.97	< 0.001	2.58**	1.39–4.80	0.003	0.77	0.39–1.49	0.431
Level of exposure to alcohol in the media in the past 7 days	Never	1.00			1.00			1.00			1.00		
	Rarely	0.50	0.12–2.08	0.338	0.51	0.16–1.66	0.263	1.77	0.56–5.54	0.329	2.10	0.76–5.78	0.151
	Sometimes	0.70	0.18–2.68	0.606	0.50	0.17–1.50	0.219	2.29	0.80–6.60	0.124	1.53	0.61–3.82	0.365
	Most of the time	0.30	0.08–1.14	0.077	0.44	0.15–1.36	0.155	2.96	0.99–8.84	0.052	3.42*	1.29–9.04	0.013
	Always/daily	0.15**	0.04–0.62	0.008	0.39	0.12–1.24	0.11	3.31*	1.07–10.24	0.037	4.00**	1.43–11.20	0.008

^a^ Odds ratio adjusted for gender, programme of study, year of study, race, age, religion, marital status, income level, living situation, sibling alcohol consumption, parental alcohol consumption, and the external exposures to alcohol. ^b^ Risk drinking according to AUDIT-C categories

* p < 0.05

** p < 0.01

*** p < 0.001

### Preferences for alcohol interventions delivery

Most participants indicated their most preferred platform to receive alcohol intervention was one-on-one conversation with a healthcare professional (n = 399) followed by WhatsApp (n = 114) and the least (n = 60) preferred choice was delivery through a phone call (Supplementary Table 3).

## Discussion

Our study found that lifetime, past 12-months and past 30-day alcohol use was common amongst undergraduate health sciences students in Johannesburg, South Africa. Similarly, we further found a high prevalence of monthly heavy episodic drinking (> 6 standard drinks of alcohol) and risky drinking. The reported alcohol use and risky drinking rates were higher compared to previous studies conducted among college students in other provinces [10, 20, 21] and African countries including Uganda, Nigeria, and Ethiopia [26–29].

Among college students, race was associated with alcohol use and risky drinking – that was different to previous reports by Young and de Klerk, 2008 [6, 20] and Peltzer and Ramlagan, 2009 [6] that found that AUD was highest in those who identified as Coloured and that could be explained by racial representation in the study population. Our study showed that the male gender was associated with alcohol use and risky drinking and this is not different from previous studies among college students that reported high rates of harmful and hazardous alcohol use among males [10, 20, 21, 30]. Disposable income increases access to alcohol use, as such students reporting an income higher than the median for the group had higher odds of reporting alcohol use – similar to previous work among students in South Africa [6, 10, 31] and Nigeria [27].

This study underscores the importance of parenting and siblings on alcohol use at the family level. As such having a sibling and parents who used alcohol was associated with high odds of reporting alcohol use [3, 10, 20, 32–34] and that could be explained by the social learning process in which children of parents who adopt norms favourable to alcohol use imitate their parents, as such behaviours are seen as normative to the children, subsequently socially reinforced, and are thus also adopted by them [35]. However, Mahedy et al., 2018 found an indirect effect between parental alcohol use and children’s alcohol use in adulthood through the mediators associating with deviant peers and early alcohol initiation [36]. Due to the strong ties shared by siblings, and their role model effect, siblings exert their influence for alcohol use mainly through the peer domain. They may also act as alcohol use advocacy agents thus not only encouraging and approving its use, but also supplying the alcohol to their siblings [35].

Exposure to alcohol marketing in the media (including social media) in the past 7 days, especially always/daily, carried greater risk for ongoing and risky alcohol use as it was associated with use in the past month, and risky drinking. This influence by the alcohol industry on risky drinking is evident in previous studies by Young and de Klerk, 2008 [20] and Francis et al., 2014 [3]. Similarly, Engels et al., 2009 found that watching a movie that portrays scenes of alcohol use led study participants to drink higher levels of alcohol while watching the movie – with those exposed to such scenes drinking an average of 1.5 glasses more than individuals not exposed to such scenes whether in movies or commercials [37]. A systematic review and study by Gupta. et al., 2016 & Gupta et al., 2018 also supports the finding that being exposed to alcohol-related content through the internet was associated with individuals’ alcohol use [38, 39]. The term to describe this effect is “cultures of intoxication” in which such exposure creates an active pathway that promotes alcohol use and risky drinking with positivity resulting in such behaviours by the viewer [38]. However, always/daily exposure to alcohol was associated with lower lifetime alcohol use. This is contrary to findings from a systematic review that found that exposure to alcohol through media was associated with the initiation of alcohol consumption [40].

The effect of religiosity on alcohol use in high school students has been previously established – it was found that an inverse relationship between learners with high religiosity levels and the use of alcohol in the last 30 days existed [41]. In our study being a member of the Muslim religion was associated with greatly reduced odds of lifetime and past 12-month alcohol use. These results are supported by previous studies that found similar results. One study found that being Muslim was negatively associated with alcohol use, specifically in adolescent boys [14]. This reflects a subculture that promotes abstinence from alcohol use through strictly forbidding the use of alcohol within the religion – which is achieved by Muslims communicating more explicit messages regarding alcohol. This contributes to the spiritual convictions regarding their stance on alcohol use, encouraging them to drink less or even none at all [12].

One-on-one physical sessions with a healthcare professional was identified as the most acceptable modality through which to receive alcohol and other health-related interventions, followed by cellular communications through WhatsApp or mobile phone Short Messages (SMS). A study by Johannson et. el., 2021 also found that the preferred mode of intervention was the face-to-face one, with more participants engaging in this mode than the internet-based ones [17]. Contrary to this, other studies conducted in SSA on patients, pregnant women, sex workers, and students found that the most common and preferred intervention for AUD were online therapy programmes such as Screening, Brief Intervention, and Referral to Treatment [16, 42]. This offers insight to potential platforms of delivery in the implementation of health promotion or therapeutic interventions such as digital therapeutics.

The findings of this study should be interpreted in light of the potential limitations. The response rate was low 631/4016 (15.7%) but in keeping with expected response rates for online surveys [43]. However, our sensitivity analysis for this potential selection bias due to non-response showed that there was minimal difference between participants who completed the surveys and those who did not. There was no notable difference between respondents and non-respondents among all the population characteristics except or ‘living situation’ – where those living alone were significantly less likely to respond to the survey Supplementary Table 2). These findings are supported by a previous study that also found that individuals living alone were less likely to participate in their surveys [44]. Also, selection bias may have been due to responses received by volunteers and participants who responded to the survey may have differed from those who did not. Unfortunately, data on this was not collected thus we cannot comment on that aspect. Second, social desirability bias due to self-reporting of alcohol outcomes, however, this bias was largely reduced by anonymous and self-administration of the surveys. Third, part of the study was conducted during the 2020 COVID-19 pandemic lockdown, during which the alcohol sales were restricted this may have led to low reported alcohol use in the last 30 days. Fourth, because of the cross-sectional nature of the study it was not possible to assess causality whether the risk factors caused the outcomes. Lastly, the findings could only be generalized to health sciences university students in Johannesburg, South Africa.

## Conclusion

In conclusion, reported alcohol use and risky drinking are common among undergraduate university students at the University of the Witwatersrand in Gauteng South Africa. Alcohol use and risky drinking were associated with sociodemographic factors and exposure to alcohol adverts. There is an urgent need to design and pilot interventions to address harmful alcohol use among college/university students. The students indicated face to face meeting with healthcare providers as their most preferred approach to receive interventions. While WhatsApp messaging was rated as a second choice these should be investigated further, particularly with regard to feasibility and effectiveness. Future studies should further explore in-depth the impact of media on alcohol consumption and evaluate the feasibility of integrating SBIRT provided by trained providers among undergraduate university students.

## Electronic supplementary material

Below is the link to the electronic supplementary material.

Supplementary Material 1 Findings of bivariate analysis on factors associated with alcohol-use and risky drinking among study participants

## Data Availability

The datasets used and/or analysed during the current study are available from the corresponding author on reasonable request.

## Abbreviations

aOR: Adjusted Odds Ratio
AUD: Alcohol Use Disorder
AUDIT-C: Alcohol Use Disorder Identification Test
COVID-19: Coronavirus Disease
DALYs: Disability-Adjusted Life Years
FHS: Faculty of Health Sciences
MBBCh: Bachelor of Medicine and Bachelor of Surgery Degree
REDCap: Research Electronic Data Capture
SMS: Short Message Service
SSA: Sub-Saharan Africa
USD: United States Dollar
WHO: World Health Organisation
WITS: University of the Witwatersrand
