# Supplementary material for: Prevalence and correlates of alcohol use and risky drinking among undergraduate students in Johannesburg, South Africa: a cross-sectional study
Source: BMC Psychiatry. 2023 Aug 1;23:553. doi: 10.1186/s12888-023-05043-w (PMC10394774; doi:10.1186/s12888-023-05043-w)
Supplement: Supplementary file 1 — Supplementary Material 1 Findings of bivariate analysis on factors associated with alcohol-use and risky drinking among study participants [file 12888_2023_5043_MOESM1_ESM.docx]

Supplementary Tables

**Supplementary Table 1: Findings of bivariate analysis on factors associated with alcohol-use and alcohol-use-disorder among study participants**

| Characteristic | Categories | Ever Use | | | | | Alcohol use in last 12 months | | | | | Alcohol use in last 30 days | | | | | Risky drinking (AUD) | | | | |
| --- | --- | --- | --- | --- | --- | --- | --- | --- | --- | --- | --- | --- | --- | --- | --- | --- | --- | --- | --- | --- | --- |
|  |  | No | | Yes | | P-value | No | | Yes | | P-value | No | | Yes | | P-value | No | | Yes | | P-value |
|  |  | n | % | n | % |  | n | % | n | % |  | n | % | n | % |  | n | % | n | % |  |
|  |  |  |  |  |  |  |  |  |  |  |  |  |  |  |  |  |  |  |  |  |  |
| Gender | Female | 69 | 18.4 | 305 | 81.6 |  | 105 | 28.1 | 269 | 71.9 |  | 232 | 62.0 | 142 | 38.0 |  | 122 | 45.7 | 145 | 54.3 |  |
|  | Male | 43 | 26.4 | 120 | 73.6 |  | 55 | 33.7 | 108 | 66.3 |  | 106 | 65.0 | 57 | 35.0 |  | 48 | 44.0 | 61 | 56.0 |  |
|  | Total | 112 | 20.9 | 425 | 79.1 | 0.038 | 160 | 29.8 | 377 | 70.2 | 0.187 | 338 | 62.9 | 199 | 37.1 | 0.508 | 170 | 45.2 | 206 | 54.8 | 0.770 |
|  |  |  |  |  |  |  |  |  |  |  |  |  |  |  |  |  |  |  |  |  |  |
| Training programme | MBCHB | 76 | 20.5 | 294 | 79.5 |  | 115 | 31.1 | 255 | 68.9 |  | 224 | 60.5 | 146 | 39.5 |  | 112 | 43.8 | 144 | 56.3 |  |
|  | Other | 36 | 21.4 | 132 | 78.6 |  | 45 | 26.8 | 123 | 73.2 |  | 115 | 68.5 | 53 | 31.5 |  | 58 | 47.9 | 63 | 52.1 |  |
|  | Total | 112 | 20.8 | 426 | 79.2 | 0.814 | 160 | 29.7 | 378 | 70.3 | 0.312 | 339 | 63.0 | 199 | 37.0 | 0.078 | 170 | 45.1 | 207 | 54.9 | 0.446 |
|  |  |  |  |  |  |  |  |  |  |  |  |  |  |  |  |  |  |  |  |  |  |
| Current year of study | 1 | 29 | 27.9 | 75 | 72.1 |  | 35 | 33.7 | 69 | 66.3 |  | 74 | 71.2 | 30 | 28.8 |  | 32 | 46.4 | 37 | 53.6 |  |
|  | 2 | 14 | 17.7 | 65 | 82.3 |  | 22 | 27.8 | 57 | 72.2 |  | 56 | 70.9 | 23 | 29.1 |  | 26 | 47.3 | 29 | 52.7 |  |
|  | 3 | 24 | 20.7 | 92 | 79.3 |  | 38 | 32.8 | 78 | 67.2 |  | 75 | 64.7 | 41 | 35.3 |  | 41 | 52.6 | 37 | 47.4 |  |
|  | 4 | 25 | 21.4 | 92 | 78.6 |  | 37 | 31.6 | 80 | 68.4 |  | 76 | 65.0 | 41 | 35.0 |  | 37 | 45.7 | 44 | 54.3 |  |
|  | 5 | 16 | 27.1 | 43 | 72.9 |  | 19 | 32.2 | 40 | 67.8 |  | 33 | 55.9 | 26 | 44.1 |  | 15 | 37.5 | 25 | 62.5 |  |
|  | 6 | 4 | 6.3 | 59 | 93.7 |  | 9 | 14.3 | 54 | 85.7 |  | 25 | 39.7 | 38 | 60.3 |  | 19 | 35.2 | 35 | 64.8 |  |
|  | Total | 112 | 20.8 | 426 | 79.2 | 0.023 | 160 | 29.7 | 378 | 70.3 | 0.110 | 339 | 63.0 | 199 | 37.0 | 0.001 | 170 | 45.1 | 207 | 54.9 | 0.417 |
|  |  |  |  |  |  |  |  |  |  |  |  |  |  |  |  |  |  |  |  |  |  |
| Racial identity | Black African | 35 | 19.1 | 148 | 80.9 |  | 66 | 36.1 | 117 | 63.9 |  | 149 | 81.4 | 34 | 18.6 |  | 65 | 56.0 | 51 | 44.0 |  |
|  | Coloured | 7 | 28.0 | 18 | 72.0 |  | 10 | 40.0 | 15 | 60.0 |  | 20 | 80.0 | 5 | 20.0 |  | 9 | 60.0 | 6 | 40.0 |  |
|  | Indian/Asian | 58 | 50.4 | 57 | 49.6 |  | 61 | 53.0 | 54 | 47.0 |  | 89 | 77.4 | 26 | 22.6 |  | 19 | 35.2 | 35 | 64.8 |  |
|  | White | 7 | 3.5 | 192 | 96.5 |  | 18 | 9.0 | 181 | 91.0 |  | 69 | 34.7 | 130 | 65.3 |  | 70 | 38.5 | 112 | 61.5 |  |
|  | Total | 107 | 20.5 | 415 | 79.5 | <0.001 | 155 | 29.7 | 367 | 70.3 | <0.001 | 327 | 62.6 | 195 | 37.4 | <0.001 | 163 | 44.4 | 204 | 55.6 | 0.006 |
|  |  |  |  |  |  |  |  |  |  |  |  |  |  |  |  |  |  |  |  |  |  |
| Age (in years) | Less than 20 years | 48 | 24.1 | 151 | 75.9 |  | 60 | 30.2 | 139 | 69.8 |  | 142 | 71.4 | 57 | 28.6 |  | 69 | 50.4 | 68 | 49.6 |  |
|  | 20-23 years | 57 | 24.1 | 180 | 75.9 |  | 80 | 33.8 | 157 | 66.2 |  | 146 | 61.6 | 91 | 38.4 |  | 65 | 41.4 | 92 | 58.6 |  |
|  | Above 24 years | 7 | 6.9 | 95 | 93.1 |  | 20 | 19.6 | 82 | 80.4 |  | 51 | 50.0 | 51 | 50.0 |  | 36 | 43.4 | 47 | 56.6 |  |
|  | Total | 112 | 20.8 | 426 | 79.2 | 0.001 | 160 | 29.7 | 378 | 70.3 | 0.032 | 339 | 63.0 | 199 | 37.0 | 0.001 | 170 | 45.1 | 207 | 54.9 | 0.286 |
|  |  |  |  |  |  |  |  |  |  |  |  |  |  |  |  |  |  |  |  |  |  |
| Religion | Christian | 36 | 11.6 | 275 | 88.4 |  | 74 | 23.8 | 237 | 76.2 |  | 194 | 62.4 | 117 | 37.6 |  | 121 | 51.5 | 114 | 48.5 |  |
|  | Moslem | 63 | 88.7 | 8 | 11.3 |  | 68 | 95.8 | 3 | 4.2 |  | 71 | 100.0 | 0 | 0.0 |  | 2 | 66.7 | 1 | 33.3 |  |
|  | Atheist/Agnostic | 5 | 5.6 | 85 | 94.4 |  | 7 | 7.8 | 83 | 92.2 |  | 38 | 42.2 | 52 | 57.8 |  | 27 | 32.1 | 57 | 67.9 |  |
|  | Other | 8 | 12.1 | 58 | 87.9 |  | 11 | 16.7 | 55 | 83.3 |  | 36 | 54.5 | 30 | 45.5 |  | 20 | 36.4 | 35 | 63.6 |  |
|  | Total | 112 | 20.8 | 426 | 79.2 | <0.001 | 160 | 29.7 | 378 | 70.3 | <0.001 | 339 | 63.0 | 199 | 37.0 | <0.001 | 170 | 45.1 | 207 | 54.9 | 0.008 |
|  |  |  |  |  |  |  |  |  |  |  |  |  |  |  |  |  |  |  |  |  |  |
| Marital status | Single | 95 | 24.4 | 295 | 75.6 |  | 129 | 33.1 | 261 | 66.9 |  | 265 | 67.9 | 125 | 32.1 |  | 123 | 47.1 | 138 | 52.9 |  |
|  | In a relationship | 17 | 11.5 | 131 | 88.5 |  | 31 | 20.9 | 117 | 79.1 |  | 74 | 50.0 | 74 | 50.0 |  | 47 | 40.5 | 69 | 59.5 |  |
|  | Total | 112 | 20.8 | 426 | 79.2 | 0.001 | 160 | 29.7 | 378 | 70.3 | 0.006 | 339 | 63.0 | 199 | 37.0 | <0.001 | 170 | 45.1 | 207 | 54.9 | 0.234 |
|  |  |  |  |  |  |  |  |  |  |  |  |  |  |  |  |  |  |  |  |  |  |
| Income (median = R2,000) | Equal to or below median | 78 | 23.9 | 248 | 76.1 |  | 110 | 33.7 | 216 | 66.3 |  | 230 | 70.6 | 96 | 29.4 |  | 105 | 48.8 | 110 | 51.2 |  |
|  | Greater than median | 34 | 16.0 | 178 | 84.0 |  | 50 | 23.6 | 162 | 76.4 |  | 109 | 51.4 | 103 | 48.6 |  | 65 | 40.1 | 97 | 59.9 |  |
|  | Total | 112 | 20.8 | 426 | 79.2 | 0.028 | 160 | 29.7 | 378 | 70.3 | 0.012 | 339 | 63.0 | 199 | 37.0 | <0.001 | 170 | 45.1 | 207 | 54.9 | 0.092 |
|  |  |  |  |  |  |  |  |  |  |  |  |  |  |  |  |  |  |  |  |  |  |
| Living arrangement | Living with an adult | 62 | 23.8 | 198 | 76.2 |  | 86 | 33.1 | 174 | 66.9 |  | 166 | 63.8 | 94 | 36.2 |  | 84 | 48.6 | 89 | 51.4 |  |
|  | Living alone | 47 | 17.7 | 219 | 82.3 |  | 70 | 26.3 | 196 | 73.7 |  | 166 | 62.4 | 100 | 37.6 |  | 82 | 41.8 | 114 | 58.2 |  |
|  | Other | 3 | 25.0 | 9 | 75.0 |  | 4 | 33.3 | 8 | 66.7 |  | 7 | 58.3 | 5 | 41.7 |  | 4 | 50.0 | 4 | 50.0 |  |
|  | Total | 112 | 20.8 | 426 | 79.2 | 0.205 | 160 | 29.7 | 378 | 70.3 | 0.229 | 339 | 63.0 | 199 | 37.0 | 0.890 | 170 | 45.1 | 207 | 54.9 | 0.416 |
|  |  |  |  |  |  |  |  |  |  |  |  |  |  |  |  |  |  |  |  |  |  |
| Have any siblings who drink alcohol | No | 75 | 41.0 | 108 | 59.0 |  | 95 | 51.9 | 88 | 48.1 |  | 151 | 82.5 | 32 | 17.5 |  | 45 | 51.1 | 43 | 48.9 |  |
|  | Yes | 27 | 8.9 | 275 | 91.1 |  | 51 | 16.9 | 251 | 83.1 |  | 155 | 51.3 | 147 | 48.7 |  | 107 | 42.8 | 143 | 57.2 |  |
|  | Total | 102 | 21.0 | 383 | 79.0 | <0.001 | 146 | 30.1 | 339 | 69.9 | <0.001 | 306 | 63.1 | 179 | 36.9 | <0.001 | 152 | 45.0 | 186 | 55.0 | 0.176 |
|  |  |  |  |  |  |  |  |  |  |  |  |  |  |  |  |  |  |  |  |  |  |
| Have any parent who drink alcohol | No | 90 | 47.4 | 100 | 52.6 |  | 114 | 60.0 | 76 | 40.0 |  | 162 | 85.3 | 28 | 14.7 |  | 35 | 45.5 | 42 | 54.5 |  |
|  | Yes | 20 | 5.9 | 320 | 94.1 |  | 40 | 11.8 | 300 | 88.2 |  | 170 | 50.0 | 170 | 50.0 |  | 135 | 45.3 | 163 | 54.7 |  |
|  | Total | 110 | 20.8 | 420 | 79.2 | <0.001 | 154 | 29.1 | 376 | 70.9 | <0.001 | 332 | 62.6 | 198 | 37.4 | <0.001 | 170 | 45.3 | 205 | 54.7 | 0.981 |
|  |  |  |  |  |  |  |  |  |  |  |  |  |  |  |  |  |  |  |  |  |  |
| Level of exposure to alcohol in the media in the past 7 days | Never | 17 | 29.3 | 41 | 70.7 |  | 22 | 37.9 | 36 | 62.1 |  | 47 | 81.0 | 11 | 19.0 |  | 24 | 64.9 | 13 | 35.1 |  |
|  | Rarely | 17 | 18.7 | 74 | 81.3 |  | 28 | 30.8 | 63 | 69.2 |  | 62 | 68.1 | 29 | 31.9 |  | 27 | 43.5 | 35 | 56.5 |  |
|  | Sometimes | 32 | 18.5 | 141 | 81.5 |  | 50 | 28.9 | 123 | 71.1 |  | 105 | 60.7 | 68 | 39.3 |  | 63 | 51.6 | 59 | 48.4 |  |
|  | Most of the time | 23 | 18.9 | 99 | 81.1 |  | 31 | 25.4 | 91 | 74.6 |  | 71 | 58.2 | 51 | 41.8 |  | 35 | 38.5 | 56 | 61.5 |  |
|  | Always/daily | 23 | 24.5 | 71 | 75.5 |  | 29 | 30.9 | 65 | 69.1 |  | 54 | 57.4 | 40 | 42.6 |  | 21 | 32.3 | 44 | 67.7 |  |
|  | Total | 112 | 20.8 | 426 | 79.2 | 0.453 | 160 | 29.7 | 378 | 70.3 | 0.538 | 339 | 63.0 | 199 | 37.0 | 0.018 | 170 | 45.1 | 207 | 54.9 | 0.008 |

**Supplementary Table 2: Characteristics of study participants who completed the survey compared to those who did not**

| Characteristics | Categories | Questionnaire Status | | | | P-value |
| --- | --- | --- | --- | --- | --- | --- |
|  |  | Incomplete | | Complete | |  |
|  |  | n | % | n | % |  |
|  |  |  |  |  |  |  |
| Age | <20 years | 31 | 33 | 199 | 37 |  |
|  | 20-23 years | 47 | 50 | 237 | 44.1 |  |
|  | ≥ 24 years | 16 | 17 | 102 | 19 | 0.564 |
|  |  |  |  |  |  |  |
| Sex | Female | 69 | 73.4 | 374 | 69.65 |  |
|  | Male | 25 | 26.6 | 163 | 30.35 | 0.462 |
|  |  |  |  |  |  |  |
| Race | Black African | 34 | 36.2 | 183 | 35.1 |  |
|  | Coloured | 1 | 1.1 | 25 | 4.79 |  |
|  | Indian/Asian | 13 | 13.8 | 115 | 22 |  |
|  | White | 46 | 48.94 | 199 | 38.1 | 0.056 |
|  |  |  |  |  |  |  |
| Training programme | MBCHB | 62 | 66 | 370 | 68.8 |  |
|  | Other | 32 | 34 | 168 | 31.2 | 0.588 |
|  |  |  |  |  |  |  |
| Current year of study | 1 | 19 | 20.2 | 104 | 19.3 |  |
|  | 2 | 14 | 14.9 | 79 | 14.7 |  |
|  | 3 | 20 | 21.3 | 116 | 21.6 |  |
|  | 4 | 21 | 22.3 | 117 | 21.8 |  |
|  | 5 | 13 | 13.8 | 59 | 11 |  |
|  | 6 | 7 | 7.5 | 63 | 11.7 | 0.857 |
|  |  |  |  |  |  |  |
| Religion | Christian | 54 | 57.5 | 311 | 57.8 |  |
|  | Moslem | 10 | 10.6 | 71 | 13.2 |  |
|  | Atheist/Agnostic | 15 | 16 | 90 | 16.7 |  |
|  | Other | 15 | 16 | 66 | 12.3 | 0.732 |
|  |  |  |  |  |  |  |
| Marital status | Single | 68 | 72.3 | 390 | 72.5 |  |
|  | In a relationship | 26 | 27.7 | 148 | 27.5 | 0.976 |
|  |  |  |  |  |  |  |
| Income level (median = R2,000) | Less than or equal to median | 58 | 65.9 | 326 | 60.6 |  |
|  | Greater than median | 30 | 34.1 | 212 | 39.4 | 0.343 |
|  |  |  |  |  |  |  |
| Living situation | Living with an adult | 58 | 62.4 | 260 | 48.3 |  |
|  | Living alone | 35 | 37.6 | 266 | 49.4 |  |
|  | Other | 0 | 0 | 12 | 2.23 | 0.024 |

**Supplementary Table 3: Preferred platforms for alcohol related interventions by Wits undergraduate FHS students, Johannesburg, South Africa, 2019-2020**

| Characteristic | Categories | Female |  | Male |  | P-value |
| --- | --- | --- | --- | --- | --- | --- |
|  |  | n | %^a^ | n | %^a^ |  |
| Preferred platforms | One-on-one with a healthcare professional | 273 | 61.6 | 126 | 67.6 | 0.157 |
|  | SMS | 64 | 14.5 | 32 | 17 | 0.410 |
|  | Phone call | 41 | 9.3 | 19 | 10.1 | 0.739 |
|  | Newsletter or pamphlet | 63 | 14.2 | 17 | 9 | 0.074 |
|  | Facebook | 30 | 6.8 | 9 | 4.8 | 0.344 |
|  | WhatsApp | 79 | 17.8 | 35 | 18.6 | 0.815 |
|  | Other | 8 | 1.8 | 8 | 4.3 | 0.073 |

^a^ Column percentage
